# Albumin Protects Against Cyclophosphamide-Induced Hemorrhagic Cystitis by Scavenging Acrolein and Reactive Oxygen Species

**DOI:** 10.3390/biom16040536

**Published:** 2026-04-03

**Authors:** Zhuheng Shi, Zhimin Mao, Yingyu Zhang, Xiaoyu Su, Rui Jiang, Yang Sui, Xin Wang, Jie Cheng, Manabu Niimi, Jianglin Fan, Jian Yao

**Affiliations:** 1 Department of the Advanced Biomedical Research, Faculty of Medicine, University of Yamanashi, Chuo City 409-3898, Japan; 2 Department of Pathology, Faculty of Medicine, University of Yamanashi, Chuo City 409-3898, Japan; 3 Guangdong Province Key Laboratory, Southern China Institute of Large Animal Models for Biomedicine, School of Pharmacy and Food Engineering, Wuyi University, Jiangmen 529020, China

**Keywords:** albumin, cyclophosphamide, acrolein, ferroptosis, cystitis, oxidative stress

## Abstract

Cyclophosphamide (CYP) is an effective chemotherapeutic, but its use is limited by hemorrhagic cystitis caused by its toxic metabolite acrolein. Acrolein, when concentrated in the urine, triggers oxidative stress, leading to urothelial inflammation and cell death. Given that albumin is the most abundant plasma protein that contains free thiol groups capable of neutralizing electrophiles and oxidants, we, therefore, hypothesized that albumin could mitigate CYP-induced bladder injury. Here, we tested this hypothesis. In CYP-induced mouse cystitis, albumin administration markedly reduced bladder enlargement, edema, and hemorrhage, effectively normalizing the bladder weight. Albumin also reduced bladder oxidative injury and preserved the expression of anti-ferroptotic proteins, including the cystine/glutamate antiporter xCT and glutathione peroxidase 4 (GPX4). In addition, albumin-treated mice showed less leakage of inflammatory protein into bladder tissue. In vitro, albumin protected urothelial cells from acrolein-induced cell death. It also significantly prevented H_2_O_2_-induced cytotoxicity. Mechanistically, albumin acted as an extracellular scavenger that preferentially reacted with acrolein and H_2_O_2_, thereby sparing cellular components from oxidative damage. Notably, oral albumin supplementation similarly attenuated CYP-induced cystitis. Furthermore, albumin administration improved survival in a high-dose CYP toxicity model. These findings establish albumin as a potent protector against CYP-induced toxicity by sequestering acrolein and scavenging reactive oxygen species. Albumin supplementation could be a practical strategy to mitigate chemotherapy-associated bladder and systemic injury.

## 1. Introduction

Cystitis is a prevalent urological condition defined by inflammation and injury of the bladder mucosa. Clinically, it manifests as a constellation of debilitating symptoms including suprapubic pain, dysuria, urinary urgency, frequency, and hematuria, all of which can significantly impair a patient’s quality of life [1,2]. While most cases of cystitis arise from infectious or idiopathic causes, the condition can also develop secondary to systemic inflammatory states [3,4], radiation exposure [5], or certain drugs [2,6,7]. Among iatrogenic causes, chemotherapy-associated cystitis is a significant clinical challenge that often leads to interruption of crucial treatment regimens [8]. This highlights the importance of elucidating molecular mechanisms and developing effective therapeutic interventions.

Cyclophosphamide (CYP) is widely used as an alkylating agent for the treatment of tumors and autoimmune diseases [9,10,11]. Despite its broad clinical use, CYP administration is frequently complicated by hemorrhagic cystitis, characterized by severe hematuria, clot retention, and intractable bladder pain [8]. CYP is a prodrug that requires hepatic cytochrome P450 metabolism to yield the therapeutic metabolite phosphoramide mustard. During the process, the toxic byproduct acrolein (ACR) is also produced [12,13]. As a highly reactive α,β-unsaturated aldehyde, ACR mediates CYP-induced urothelial toxicity. After its formation in the liver, ACR is excreted and concentrated in the urine. The deposition and accumulation of ACR in the bladder urothelium disrupt urothelial barrier integrity and increase vascular permeability, leading to edema and hemorrhage. Damage to urothelial cells also induces a local inflammatory response, exacerbating bladder injury and promoting disease progression [8,12,13,14,15,16].

Mechanistically, oxidative stress is considered to play a central role. ACR is a highly reactive molecule; it forms covalent adducts with nucleophilic sites on proteins, lipids, and small thiols such as glutathione (GSH). These interactions disrupt enzymatic functions, compromise membrane integrity, and deplete the major endogenous antioxidant GSH [17,18]. In addition to GSH depletion, ACR also stimulates the production of reactive oxygen species (ROS), impairs the generation and function of antioxidants, and propagates lipid peroxidation, thereby driving a self-propagating escalation of oxidative stress and cell damage [19,20,21]. Indeed, oxidative injury is a hallmark of CYP-induced cystitis, evidenced by the accumulation of oxidatively modified proteins and activation of pro-inflammatory signaling cascades [17,22,23]. Recent evidence also implicates ferroptosis—an iron-dependent form of regulated cell death triggered by unchecked lipid peroxidation when GSH/GPX4-dependent defenses fail—as a key contributor to urothelial cell loss in CYP-induced cystitis [24,25,26]. Collectively, these observations support a model in which ACR-initiated electrophilic stress and ROS generation converge to trigger oxidative damage, barrier disruption, and inflammatory reaction within the bladder.

Albumin (Alb), the most abundant protein in mammalian plasma, is a central regulator of extracellular homeostasis. Beyond its classical role in maintaining colloid oncotic pressure, Alb serves as a carrier for a variety of endogenous ligands and xenobiotics, modulating their distribution and bioavailability [27,28,29]. Importantly, Alb also functions as a major extracellular redox buffer. Its single free thiol (-SH) at cysteine-34 accounts for a substantial fraction of -SH groups in circulation, enabling Alb to effectively scavenge oxidants and reactive electrophiles [30,31]. Through these chemical properties, Alb may act as a “sacrificial” target for reactive species, thereby sparing critical cellular macromolecules from oxidative damage. Consistent with this role, Alb has been reported to attenuate oxidative injury and inflammatory signaling in various experimental models [32], although the precise mechanisms and contexts of this protection remain incompletely defined. In the context of CYP-induced cystitis, Alb’s reactivity toward electrophilic aldehydes is particularly interesting. Because ACR is the primary driver of urothelial damage, its sequestration by Alb should attenuate CYP cystitis.

Additionally, the antioxidative activities of Alb, especially its thiol-dependent neutralization of oxidants, could further reduce ACR-induced oxidative stress and thereby suppress downstream lipid peroxidation and ferroptotic pathways. Based on these thoughts, we speculated that Alb could effectively protect against CYP-induced cystitis. However, this hypothesis has not been tested.

In the present study, we evaluated the protective effects of Alb in a mouse model of CYP-induced hemorrhagic cystitis and explored the potential mechanism. Our results demonstrate that Alb administration markedly mitigates CYP-induced bladder injury through mechanisms involving sequestration of ACR and scavenging of oxidants. These findings suggest that Alb supplementation may be a practical and effective strategy to ameliorate the bladder toxicity and systemic side effects associated with chemotherapy.

## 2. Materials and Methods

### 2.1. Materials

CYP (cat no. PHR1404) was purchased from Sigma-Aldrich (St Louis, MO, USA), ACR (cat no. M-8015B-5031-03) was purchased from AccuStandard (New Haven, CT, USA). Bovine serum Alb (BSA, Fraction V) was sourced from Iwai Chemical Company (Tokyo, Japan). anti-(Cystine/Glutamate Antiporter) xCT (cat no. NB300-317) antibodies were purchased from Novus International Inc. (Oakville, ON, Canada). Anti-GPX4 (cat no. 52455), anti-β-actin (cat no. A5316), HRP-conjugated anti-rabbit and anti-mouse IgG (cat no.7776S or 7074S) were purchased from Cell Signaling Technology (Beverly, MA, USA). Anti-albumin polyclonal antibody was purchased from Proteintech (cat no. 16475-1-AP; Chicago, IL, USA). According to the manufacturer’s datasheet, this antibody recognizes human, mouse, rat, and goat serum albumin. We confirmed that it also cross-reacts with bovine albumin (Figure S1). Alexa 680 Fluor C2 maleimide (cat no. A20344) was obtained from Thermo Scientific (Rockford, IL, USA). DCP-Rho1 (cat no. 13194) was obtained from Cayman Chemical (Ann Arbor, MI, USA). Calcein- Acetoxymethyl (AM) and Propidium Iodide (PI) Staining Kit was sourced from Dojindo Molecular Technologies (Rockville, MD, USA). Hydrogen Peroxide (cat no. 084-07441) was purchased from Fujifilm Wako Pure Chemical Corporation (Osaka, Japan).

### 2.2. Animals and Animal Experiments

Male C57BL/6 mice (8–10 weeks old; 20–30 g) were used in this study. Animals were housed in a temperature-controlled facility under a 12 h light/12 h dark cycle with ad libitum access to standard chow and water and were acclimated for 1 week before experimentation.

For the CYP-induced cystitis model and Alb treatment with intraperitoneal injection, mice were randomly assigned to three groups: normal control, CYP, and CYP plus Alb (CYP + Alb) (*n* = 5 per group). Bovine serum Alb was administered by intraperitoneal (i.p.) injection at 1.5 g/kg every 12 h for a total of five doses. Mice in the normal control and CYP groups received volume-matched saline injections on the same schedule. Three hours after the third Alb (or saline) injection, CYP, dissolved in sterile saline at 30 mg/mL, was administered i.p. at the volume of 10 μL per gram of body weight (i.e., 300 mg/kg total). Mice were euthanized 24 h after CYP injection, and blood and bladder tissues were collected for subsequent analyses.

For the CYP-induced cystitis model and Alb treatment with oral administration, mice were assigned and treated as described above, except that Alb was delivered by oral gavage (1.5 g/kg every 12 h for five doses) instead of i.p. injection.

For the high-dose CYP survival study, mice were assigned to the same three groups (*n* = 8 per group) and treated with or without Alb. CYP was administered i.p. at 20 μL/g (600 mg/kg). These doses were chosen based on our pilot data showing that 600 mg/kg produced 100% mortality within 24 h. Survival at different groups was monitored at regular intervals until all mice had died.

### 2.3. Cell Culture

SV-HUC-1 cells (human urothelial cells) were from ATCC (Manassas, VA, USA), which were cultured in Dulbecco’s Modified Eagle Medium (DMEM)/F-12 (11320-033, Invitrogen, Carlsbad, CA, USA) containing 10% fetal bovine serum (FBS, #F8687, Sigma-Aldrich, St. Louis, MO, USA) and antimycotic solution in a humidified atmosphere at 37 °C with 5% CO_2_/95% air. For experimental purposes, the cells were cultured in the same medium supplemented with 2% FBS.

### 2.4. Western Blot

Cells were lysed using 1 × SDS lysis buffer (62.5 mM Tris–HCl, 2% SDS, 10% glycerol). Bladder tissues were weighed, minced, and homogenized in RIPA buffer containing protease inhibitor. Cells and tissue cells were further lysed by ultrasonic disruption. Micro-BCA Assay Kit (Thermo Fisher Scientific, Waltham, MA, USA) were used to determine protein concentration. Protein electrophoresis was performed on 10~12% SDS-PAGE gels, followed by transfer to PVDF membranes through a wet transfer system. Membranes were blocked with 5% non-fat dry milk or 1~3% BSA solution at room temperature for 1 h, then washed by 0.1% PBS-Tween20 (PBST) 3 times. Primary antibody was appropriately diluted and incubated with membranes for 2 h at room temperature or overnight at 4°C. After washing by PBST 3 times, membranes were incubated with HRP-conjugated secondary antibodies for 1 h. Protein bands were detected using an enhanced chemiluminescence detection system (Nacalai Tesque, Kyoto, Japan). Chemiluminescent signals were captured using a Fujifilm Image LAS-1000 analyzer (Fujifilm, Tokyo, Japan); band intensities were quantified using NIH ImageJ software, version 1.54g (https://imagej.net/ij/). The equality of loading was verified by β-actin or EZ Blue staining as an internal reference.

### 2.5. Assessment of Protein Oxidation by Detecting Carbonylation

Protein carbonylation was measured using the OxyBlot Protein Oxidation Detection Kit (EMD Millipore, Billerica, MA, USA) following the manufacturer’s protocol as we have previously described [15]. Samples were lysed in an SDS buffer (62.5 mM Tris–HCl, pH 6.8; 2% SDS; 10% glycerol) containing 1% protease inhibitor cocktail (Nacalai Tesque, Kyoto, Japan) and 50–100 mM dithiothreitol (DTT). A 5 μL aliquot of each lysate (5–10 μg protein) was mixed with 5 μL of 12% SDS and 10 μL of 2,4-dinitrophenylhydrazine (DNPH) solution. The mixture was incubated at room temperature for 15 min to denature proteins and derivatize carbonyl groups. The reaction was stopped by adding 7.5 μL of the kit’s neutralization solution. Proteins were then separated by SDS-PAGE and transferred to membranes for Western blotting as described above. Blots were probed to detect DNP-derivatized (carbonylated) proteins, and equal loading was confirmed by EZ-Blue total protein staining.

### 2.6. Determination of Hydrogen Peroxide (H_2_O_2_) Concentration

H_2_O_2_ levels were quantified using the Cayman Chemical fluorometric H_2_O_2_ detection kit (Cat. No. 600050; Ann Arbor, MI, USA) according to the manufacturer’s instructions. Native albumin (Alb), and maleimide-treated albumin (Alb-Mal) were each prepared at 6 mg/mL. Alb-Mal was generated by pre-incubating Alb with 5 mM maleimide to block free thiol groups. Each solution was mixed with 500 µM H_2_O_2_ and incubated for 1 h at room temperature. After incubation, aliquots of the reaction mixtures were transferred to a 96-well plate and the kit’s assay and enzyme buffers were added. Fluorescence was measured on a SpectraMax GEMINI EM microplate reader (Molecular Devices, Sunnyvale, CA, USA) using 530 nm excitation and 590 nm emission wavelengths.

### 2.7. Assessment of Cell Viability with WST Reagent

Cell viability was determined using a WST assay kit (Dojindo, Kumamoto, Japan) following the manufacturer’s instructions. Cells were seeded at 1 × 10^4^ cells per well in 96-well plates. When cells reached ~80–90% confluence, they were treated as indicated. After the treatment period, WST reagent was added to each well, and cells were incubated for 30 min at 37 °C. The optical density (OD) of each well was then measured at 450 nm using a microplate reader (SpectraMax 340; Molecular Devices, Sunnyvale, CA, USA). Cell viability was calculated as the percentage of the OD of treated samples relative to that of the untreated control group.

### 2.8. Lactate Dehydrogenase (LDH) Release Assay

LDH release was measured using the LDH Cytotoxicity Detection Kit (TaKaRa Biomedicals, Otsu, Japan) following the manufacturer’s protocol. Cells were seeded at 1 × 10^4^ cells per well in 96-well plates and cultured to ∼80–90% confluence before treatment. After treatment, culture supernatants were collected for assay. Equal volumes of supernatant and the kit’s reaction buffer were mixed and incubated at room temperature for 30 min to develop color. The absorbance of the resulting solution was measured at 490 nm using a microplate reader (SpectraMax 340; Molecular Devices, Sunnyvale, CA, USA). LDH release was calculated as a percentage of total LDH, where total was determined by lysing parallel cells with 2% Triton X-100 (10 min) and measuring LDH in these lysates.

### 2.9. Calcein-AM/Propidium Iodide (PI) Staining

Live and dead cells were distinguished using the Calcein AM/PI Double Staining Kit (Dojindo, Kumamoto, Japan). Cells in 96-well plates were treated as specified, then incubated for 10–20 min at room temperature with a staining solution containing 2 mM Calcein AM and 1 μg/mL propidium iodide (PI). Fluorescence images were acquired on an Olympus IX71 microscope (Tokyo, Japan): live cells fluoresce green (Calcein AM-positive) and dead cells fluoresce red (PI-positive).

### 2.10. Determination of Free Thiol (-SH) Groups with Maleimide-Labeling Assay

The free thiol (-SH) content of albumin was assessed using Alexa Fluor 680 C2 maleimide labeling. Native or H_2_O_2_-oxidized albumin samples were incubated with 5 μM Alexa Fluor 680 C2 maleimide at 37 °C for 15 min. After labeling, proteins were separated by SDS-PAGE. Fluorescently labeled protein bands in the gels were then detected using a Fujifilm LAS-4000 imaging system (Fujifilm, Tokyo, Japan).

### 2.11. Determination of Sulfenic Acid (-SOH)

The detection of -SOH formation in BSA and H_2_O_2_-modified BSA was performed as previously described [33]. Briefly, BSA (10–30 μg) was incubated with 5 μM DCP Rho1 at 37 °C for 20 min to label the -SOH groups. The labeled samples were then mixed with SDS sample buffer. Western blot analysis was performed to detect the labeled samples. The fluorescence signals of DCP Rho1 were directly captured using a Fujifilm Image LAS 1000 analyzer (Fujifilm, Tokyo, Japan).

### 2.12. Histology Analysis

Tissues were fixed in 10% neutral buffered formalin, dehydrated through graded ethanol, embedded in paraffin, mounted on glass slides, and stained with hematoxylin and eosin (HE) using standard protocols.

### 2.13. Statistical Analysis

Data are expressed as mean ± standard error (SE). Comparisons between two groups were performed using Student’s *t*-test. For analyses involving multiple groups sharing a common control, one-way or two-way analysis of variance (ANOVA) was applied as appropriate. Survival curves were generated using the Kaplan–Meier method. Differences in survival among groups were evaluated by the log-rank test. All statistical analyses were conducted using Microsoft Excel (Microsoft, Redmond, WA, USA) or GraphPad Prism 9 software (version 9; GraphPad Software, San Diego, CA, USA). The value of *p* < 0.05 was considered statistically significant.

## 3. Results

### 3.1. Alb Attenuates CYP-Induced Cystitis

**Figure 1:**
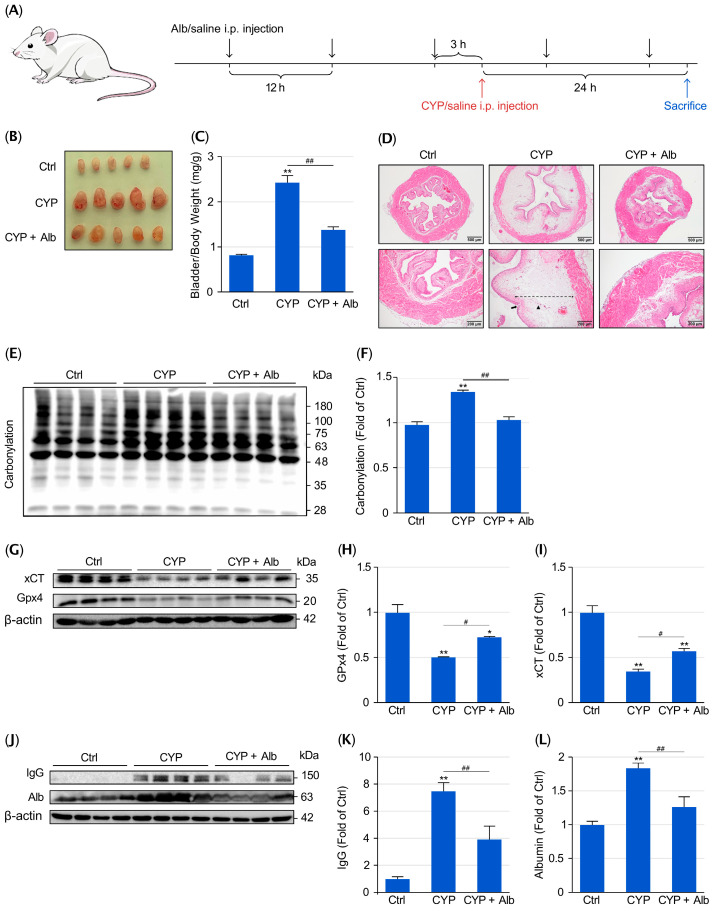
Alb attenuates CYP-induced cystitis. (**A**) Experimental design. Mice were randomized into three groups: normal control (Ctrl), CYP, and CYP + Alb. Alb was administered intraperitoneally (1.5 g/kg) every 12 h for a total of five doses. CYP (300 mg/kg, i.p.) was given 3 h after the third Alb injection. Mice were euthanized 24 h after CYP administration; bladders were harvested, photographed, weighed, and processed for downstream analyses. (**B**) Representative gross bladder images from Ctrl, CYP, and CYP + Alb groups. CYP induced marked bladder swelling and hemorrhage, which were reduced by Alb treatment. (**C**) Bladder weight-to-body weight ratio. Data are mean ± SE (*n* = 5). ** *p* < 0.01 vs. Ctrl; ## *p* < 0.01. (**D**) Representative H&E staining of bladder sections. Arrows indicate erosion/disruption of the urothelial layer; dashed lines indicate submucosal thickening/edema; arrowheads indicate inflammatory cell infiltration. (**E**,**F**) Bladder protein oxidation assessed by protein carbonylation. (**E**) Representative blots; (**F**) Densitometric quantification (mean ± SE; *n* = 4). (**G**–**I**) Ferroptosis-related proteins GPX4 and xCT in bladder lysates assessed by Western blotting. (**G**) Representative immunoblots; (**H**,**I**) Densitometric quantification. (**J**–**L**) IgG and Alb abundance in bladder protein extracts (expressed relative to Ctrl) as indices of vascular leakage/extravasation, assessed by Western blotting. Data are mean ± SE (*n* = 4). * *p* < 0.05, ** *p* < 0.01 vs. Ctrl; # *p* < 0.05, ## *p* < 0.01. Of note, IgG and albumin abundance in bladder lysates are used as indices of vascular protein leakage.

To determine whether Alb protects against CYP-induced cystitis, mice were intraperitoneally administered Alb before receiving a single injection of CYP. Bladder injury was evaluated 24 h after CYP exposure (Figure 1A). As expected, CYP-treated mice developed typical features of hemorrhagic cystitis, including marked bladder enlargement, edema, and mucosal hemorrhage. Alb pretreatment, however, markedly reduced these pathological changes (Figure 1B). In line with the macroscopic findings, CYP administration caused a significant increase in the bladder weight-to-body weight ratio, indicating the existence of tissue edema and inflammation. In contrast, Alb treatment greatly prevented the increase (Figure 1C). These results thus indicate that Alb mitigated bladder edema and overall injury.

HE staining of the bladder further confirmed the protective effects of Alb. CYP-induced loss and erosion of the epithelial layer, thickening of the subepithelial layer, and inflammatory cell infiltration, as revealed by HE staining, were markedly prevented by Alb (Figure 1D).

As Oxidative stress is considered to play a central role in CYP-induced bladder damage [17,22], we assessed protein carbonylation, a widely used marker of oxidative stress [25]. Figure 1E,F show that CYP treatment led to a pronounced increase in protein carbonylation in the bladder, which could be largely prevented by Alb, suggesting that Alb attenuates CYP-induced oxidative stress in vivo. Because ferroptosis is a downstream event of oxidative stress and has been reported to contribute to CYP-induced cystitis [26], we, therefore, examined the levels of key ferroptosis-regulating proteins. In CYP-treated bladders, the levels of the cystine transporter xCT and the lipid-peroxide repairing enzyme GPX4 were significantly reduced, indicating the existence of ferroptotic cell injury. Alb treatment, however, largely preserved the levels of xCT and GPX4 (Figure 1G–I), indicating that Alb preserved the anti-ferroptotic defenses and protected the bladder from ferroptotic cell death.

Inflammation-related vascular leakage, leading to edema and extravasation of plasma proteins into the bladder tissue, is another feature of CYP-induced cystitis. Consistently, bladders from CYP-treated mice showed increased extravasation of circulating proteins, as indicated by the accumulation of IgG and Alb in bladder tissue (Figure 1J–L). Alb treatment greatly reduced the protein leakage, implying that Alb suppressed the inflammatory response and preserved vascular barrier integrity. Notably, the albumin band detected in the BSA-treated group contains both endogenous mouse albumin and the administered bovine albumin, since our anti-albumin antibody cross-reacts with both species (Supplementary Figure S1). Nevertheless, the combined albumin signal (mouse + bovine) in bladder tissue was lower in BSA-treated mice than in the CYP-only control. This suggests that albumin treatment potently reduced vascular leakage of serum albumin, indicating enhanced vascular integrity. Collectively, these results demonstrate that Alb substantially protects mice from CYP-induced bladder injury.

### 3.2. Alb Prevents ACR- and H_2_O_2_-Induced Urothelial Cell Death In Vitro

**Figure 2:**
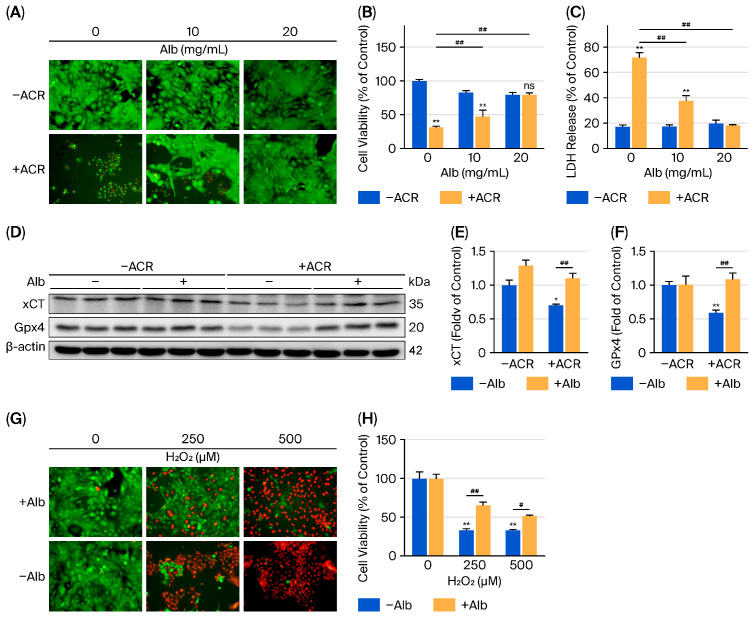
Alb prevents urothelial cell injury in vitro. (**A**–**C**) Alb protects against ACR (ACR)-induced cytotoxicity. Cultured urothelial cells were treated with ACR (100 µM) in the presence or absence of Alb at the indicated concentrations. Cell injury/viability was assessed by AM/PI live–dead staining (**A**), formazan formation assay (**B**), and lactate dehydrogenase (LDH) release (**C**). Data are mean ± SE (*n* = 3). ** *p* < 0.01 vs. untreated control; ## *p* < 0.01 vs. ACR alone. (**D**–**F**) Alb preserves ferroptosis-defense proteins following ACR exposure. Cells were treated with ACR (50 µM) with or without Alb (30 mg/mL). After 9 h, cell lysates were analyzed by Western blotting for GPX4 and xCT. (**D**) Representative immunoblots; (**E**,**F**) Densitometric quantification. Data are mean ± SE (*n* = 3). * *p* < 0.05, ** *p* < 0.01 vs. −ACR control; # *p* < 0.05 and ## *p* < 0.01 vs. ACR alone (as indicated). (**G**,**H**) Alb protects against H_2_O_2_-induced injury. Cells were treated with the indicated concentrations of H_2_O_2_ in the presence or absence of Alb (30 mg/mL). Cell injury/viability was assessed using the same assays as in (**A**–**C**).

Given that the CYP metabolite ACR mediates its toxic effect in the bladder [25], we therefore tested whether Alb could directly protect urothelial cells from ACR toxicity. For this purpose, cultured urothelial cells were exposed to ACR in the presence or absence of Alb, and cell damage was examined. As expected, ACR caused urothelial cell death, evidenced by markedly decreased Calcein-AM staining of living cells, the appearance of PI-positive red dead cells in Calcein-AM/PI staining, reduced MTT formazan formation, and a significant increase in LDH release. In the presence of Alb, however, these cytotoxic effects of ACR were largely prevented. Alb treatment preserved Calcein-AM–positive viable cells, maintained normal levels of formazan production, and prevented the LDH release (Figure 2A–C). Furthermore, consistent with the in vivo occurrence of ferroptotic responses, ACR also downregulated xCT and GPX4 in cultured urothelial cells. These changes could also be largely prevented by Alb (Figure 2D–F).

Because ACR toxicity is tightly linked to ROS generation and oxidative stress [22], we therefore determined whether Alb also protects cells against oxidant-induced injury. For this purpose, we chose hydrogen peroxide (H_2_O_2_) to induce oxidative injury in urothelial cells. As a major oxidant with membrane-permeable ability, H_2_O_2_ has been previously shown to be critically implicated in CYP cystitis [34,35]. Exposure to H_2_O_2_ caused significant cell death, as indicated by an obvious disappearance of Calcein-positive living cells in Calcein-AM/PI co-staining, along with the decreased formazan formation in MTT viability assay and the increased LDH release. When Alb was present during H_2_O_2_ exposure, these changes were significantly prevented (Figure 2G,H). Thus, Alb directly protects urothelial cells from ACR and oxidant-induced cytotoxicity.

### 3.3. Alb Diverts Oxidative Damage by Preferentially Reacting with ACR and H_2_O_2_

**Figure 3:**
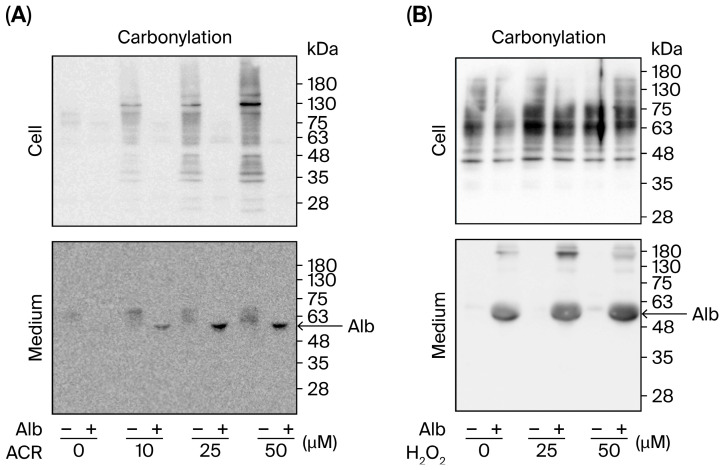
Alb diverts ACR- and H_2_O_2_-induced oxidative modification from cellular proteins to extracellular proteins. Cultured urothelial cells were exposed to the indicated concentrations of ACR (**A**) or H_2_O_2_ (**B**) in the presence or absence of Alb (30 mg/mL) for 9 h. Protein carbonylation was assessed in cell lysates (**upper panels**) and culture supernatants (**lower panels**) using an OxyBlot assay. ACR and H_2_O_2_ induced a concentration-dependent increase in protein carbonylation in cellular lysates, which was markedly reduced by Alb co-treatment. In parallel, Alb co-treatment increased carbonylated protein signal in the supernatants, with a dominant band in the ~48–63 kDa range consistent with Alb (arrow).

Given that Alb has a free cysteine in its structure, it may preferably react with ACR and H_2_O_2_, thereby diverting ACR and H_2_O_2_ away from damaging cellular components. To test this possibility, we examined protein carbonylation in both cell lysates and culture media after ACR or H_2_O_2_ exposure, with or without Alb. Figure 3A,B show that exposure of cells to ACR or H_2_O_2_ resulted in a concentration-dependent increase in protein carbonylation across a broad range of molecular weights in cell lysates, consistent with widespread oxidative modification of cellular proteins. However, when cells were co-treated with Alb, the rise in cellular protein carbonylation was minimal. Correspondingly, a substantial increase in protein carbonylation was detected in the culture media from Alb co-treated samples, which was predominantly located at ~66 kDa, consistent with Alb. This inverse relationship (low cellular vs. high extracellular protein oxidation in the presence of Alb) supports the idea that Alb preferentially reacted with ACR and H_2_O_2_ in the extracellular environment, thus sparing cellular proteins from oxidative damage.

**Figure 4:**
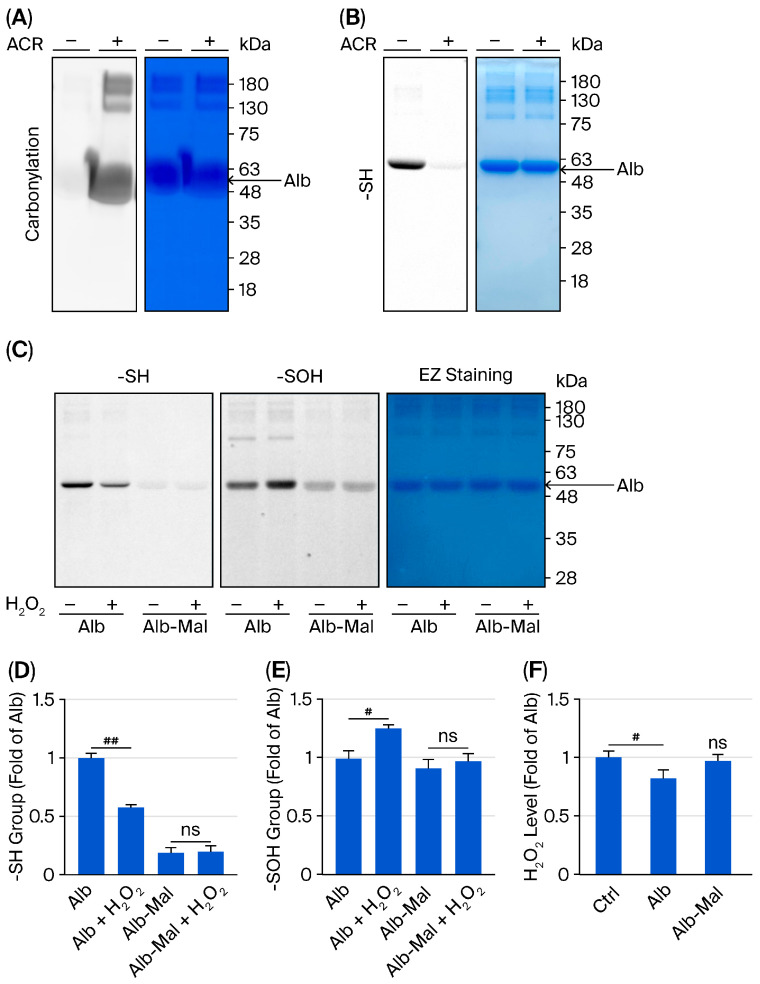
Alb directly reacts with ACR and consumes H_2_O_2_ via a thiol-dependent mechanism. (**A**,**B**) ACR modifies Alb. Alb was incubated with ACR at room temperature for 1 h. Alb carbonylation (**A**) and free thiol (-SH) content (**B**) were assessed using an OxyBlot assay and a maleimide-labeling assay, respectively. Co-incubation with ACR increased Alb carbonylation and decreased Alb free -SH content. Equal loading was confirmed by EZ Blue staining of the membrane/gel. (**C**–**E**) Thiol-dependent interaction of Alb with H_2_O_2_. Alb (6 mg/mL) or thiol-blocked Alb pretreated with maleimide (Alb–Mal; 6 mg/mL) was incubated with H_2_O_2_ (500 µM) at room temperature for 1 h. Thiol redox status was assessed by measuring-SOH formation and remaining -SH groups (**C**). Densitometric quantification is shown in (**D**,**E**). (**F**) Alb reduces H_2_O_2_ levels in a thiol-dependent manner. Residual H_2_O_2_ concentration was measured after incubation with Alb or Alb–Mal. Data are mean ± SE (*n* = 3). ## *p* < 0.01; # *p* < 0.05; ns, not significant.

To directly test whether Alb reacts with ACR, Alb was incubated with ACR in a cell-free system, and Alb modification was assessed. Co-incubation with ACR markedly increased Alb carbonylation (Figure 4A,B), consistent with covalent ACR–Alb adduct formation.

We next examined whether Alb directly neutralizes H_2_O_2_ through its thiol group. Alb was pretreated with or without the thiol-blocking reagent maleimide and then exposed to H_2_O_2_. Thiol redox status was assessed by quantifying -SH groups using a maleimide-labeling assay and sulfenic acid formation (-SOH) using the dimedone-based probe DCP. As shown in Figure 4C–E, H_2_O_2_ exposure caused a pronounced loss of Alb free thiols, accompanied by increased -SOH formation, indicating thiol oxidation. This reaction was associated with a significant reduction in H_2_O_2_ concentration (Figure 4F). Importantly, these effects were abolished when Alb thiols were blocked by maleimide, demonstrating thiol dependence.

Collectively, these findings support a mechanism in which Alb preferentially reacts with toxic electrophile ACR and oxidant H_2_O_2_ in the extracellular space, thereby diverting these reactive molecules away from cellular targets and limiting intracellular oxidative injury and cell death.

### 3.4. Oral Administration of Alb Attenuates CYP-Induced Cystitis

**Figure 5:**
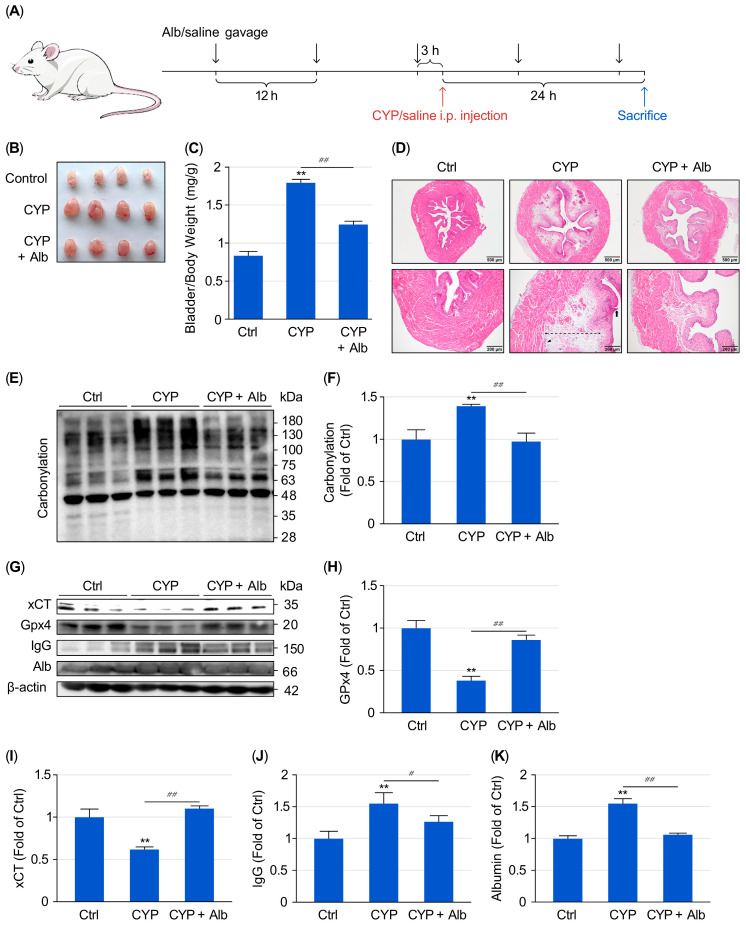
Oral Alb administration attenuates CYP-induced cystitis. (**A**) Experimental design. Mice received bovine serum Alb (1.5 g/kg) or vehicle by oral gavage every 12 h for a total of five doses. CYP (300 mg/kg, i.p.) was administered 3 h after the third Alb dose. Bladders were collected 24 h after CYP injection for analysis. (**B**) Representative gross images of bladders from control (Ctrl), CYP, and CYP + Alb groups. (**C**) Bladder weight-to-body weight ratio. Data are mean ± SE (*n* = 3). ** *p* < 0.01 vs. Ctrl; ## *p* < 0.01. (**D**) Representative H&E staining of bladder sections. Arrows indicate erosion/disruption of the urothelial layer; dashed lines indicate submucosal thickening/edema; arrowheads indicate inflammatory cell infiltration. (**E**,**F**) Bladder protein oxidation assessed by protein carbonylation. Representative blots and quantification are shown. (**G**–**K**) Bladder injury and inflammation markers. GPX4, xCT, IgG, and Alb levels in bladder lysates were assessed by Western blotting. (**G**) Representative immunoblots; (**H**,**J**,**K**) Densitometric quantification. Data are mean ± SE (*n* = 3). ** *p* < 0.01 vs. Ctrl; # *p* < 0.05 and ## *p* < 0.01.

To evaluate the translational potential of Alb as a protective agent, we tested whether oral administration of Alb could also mitigate CYP-induced cystitis. Alb was administered via gavage instead of intraperitoneal injection, and changes 24 h after CYP injection were analyzed (Figure 5A). Remarkably, oral Alb supplementation reduced CYP-induced bladder injury in a way similar to intraperitoneal Alb. Mice receiving Alb by oral gavage showed less bladder edema and hemorrhage upon CYP treatment, as assessed by gross morphology and histological analysis (Figure 5B–K). Consistently, the treatment also attenuated bladder oxidation, inflammation, and cell injury, as revealed by carbonyl formation, ferroptotic markers, and vascular leakage. These results closely mirrored those obtained with the intraperitoneal Alb injection, suggesting that Alb’s detoxifying capacity can be achieved through dietary supplementation.

### 3.5. Alb Improves Survival in a High-Dose CYP Toxicity Model

**Figure 6:**
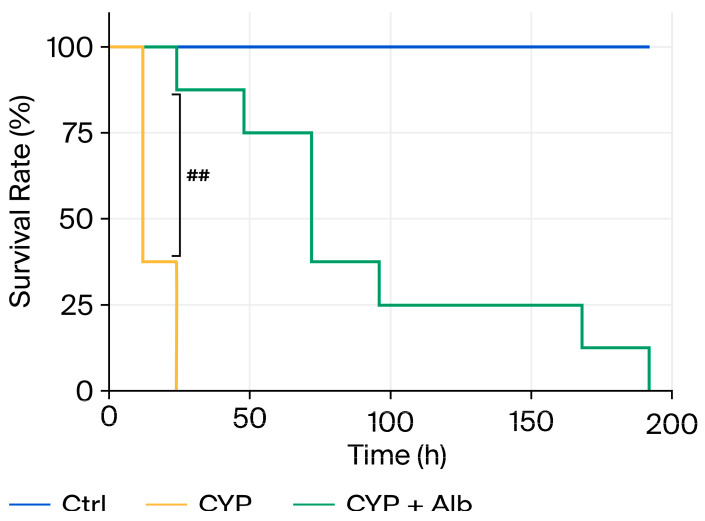
Alb improves survival in a high-dose CYP model. Mice were randomized into three groups (Ctrl, CYP, and CYP + Alb; *n* = 8 per group) and treated with Alb as described previously. CYP (600 mg/kg, i.p.) was administered, and survival was monitored every 12 h thereafter. Kaplan–Meier survival curves were generated in GraphPad Prism, and group differences were analyzed using the log-rank (Mantel–Cox) test. ## *p* < 0.01. Alb treatment significantly prolonged survival compared with CYP alone.

Finally, we investigated whether survival could improve under conditions of severe CYP toxicity. For this purpose, high-dose CYP (600 mg/mL) was used to cause systemic toxicity and mortality. Figure 6 shows that CYP-treated mice were all dead within 20 h post the injection. Strikingly, mice pretreated with Alb displayed markedly extended median lifespan (10 h in CYP vs. 72 h in Alb-treated). Most of the mice survived well beyond the 20 h time point, at which all CYP mice died. This outcome indicates that Alb may not only protect against localized bladder damage but also mitigate systemic toxic effects of high-dose CYP, thereby improving overall survival.

## 4. Discussion

This study characterized Alb as a potent protector against CYP-induced hemorrhagic cystitis and against ACR- and H_2_O_2_-driven urothelial injury. Mechanistically, Alb directly reacted with two key toxic molecules implicated in this model. It is bound to the electrophilic CYP metabolite ACR to form covalent adducts, and it scavenges the major oxidant H_2_O_2_ through thiol-dependent redox chemistry. By limiting the availability of these reactive species, Alb reduced their ability to initiate oxidative stress and downstream injury signaling, thereby attenuating bladder oxidative damage, inflammation, and tissue disruption.

In vivo, Alb treatment markedly improved the gross and histopathologic features of CYP cystitis, accompanied by reduced protein oxidation and inflammation. Importantly, Alb also preserved the expression of GPX4 and xCT, suggesting maintenance of endogenous defenses that restrain lipid peroxidation and ferroptosis-associated injury during cystitis. These findings indicate that Alb does not merely reduce visible pathology but also disrupts molecular pathways critical to urothelial defense. Our cell culture results confirmed and strengthened these conclusions. ACR, the principal urotoxic metabolite of CYP [12], caused robust urothelial cytotoxicity, which Alb significantly mitigated. Consistent with preservation of anti-ferroptotic capacity, Alb also prevented the ACR-induced loss of xCT and GPX4. Moreover, Alb reduced oxidant H_2_O_2_-induced cell injury, indicating that its cytoprotective effect is not limited to ACR but extends to diffusible oxidants. Together, the in vivo and in vitro data support a model in which Alb functions as an extracellular chemical buffer that intercepts electrophiles and oxidants upstream of cellular damage.

It is well recognized that ACR is the chief instigator of CYP-induced cystitis. ACR is generated in the liver during CYP metabolism and is excreted into the urine, where it directly contacts the bladder mucosa. Prior studies have shown that ACR reproduces the pathological features of hemorrhagic cystitis when administered to experimental animals, whereas the primary anticancer metabolite (phosphoramide mustard) does not damage the bladder [12]. Clinically, the importance of ACR is underscored by the use of mesna (2-mercaptoethane sulfonate) in cancer patients. Mesna provides free thiol groups that bind and inactivate ACR in the urinary tract, dramatically reducing the incidence of hemorrhagic cystitis without affecting CYP’s anticancer action [36]. These observations indicate that neutralizing or scavenging ACR is a highly effective strategy to prevent bladder toxicity. Our findings align well with this strategy. Alb may function as an endogenous analog of mesna, capturing ACR through its reactive thiol and other nucleophilic sites. Given the abundance of Alb in body fluids, Alb plays an important role in mitigating the harmful effects of ACR on the bladder. By targeting ACR, Alb exerts its protective action by directly addressing the cause of bladder injury rather than only its downstream consequences.

Oxidative stress is a central mechanism behind ACR cytotoxicity and CYP-induced bladder pathology [15,37]. ACR exposure rapidly depletes cellular GSH and other antioxidants, impairs key antioxidant enzymes, and provokes excessive ROS production, including superoxide and H_2_O_2_. These effects, when combined, result in widespread oxidative damage to proteins, lipids, and DNA, as well as the activation of inflammatory signaling cascades. Consistent with this understanding, numerous antioxidant compounds (including thiol donors, free radical scavengers, and iron chelators) have shown efficacy in experimental models of CYP cystitis. For instance, supplementation with antioxidants like *N*-acetylcysteine, melatonin, or plant-derived polyphenols has been reported to alleviate bladder inflammation and histological damage in CYP-treated rodents [25,32,38,39]. These interventions aim to neutralize ROS or bolster cellular antioxidant capacity, thereby interrupting the chain of oxidative injury. In our study, Alb achieved a similar outcome, evidenced by lower protein oxidation and improved cell survival, via mechanisms that directly quench both the instigating electrophile ACR and the key oxidant H_2_O_2_.

Among various forms of cell death associated with oxidative stress, ferroptosis has emerged as a particularly relevant pathway in CYP-induced bladder injury. Ferroptosis is driven by iron-dependent lipid peroxidation when glutathione is depleted, and GPX4 is compromised. Prior reports have implicated ferroptosis in the pathogenesis of hemorrhagic cystitis, noting that ferroptosis inhibitors can reduce urothelial damage in CYP models [25]. ACR is closely tied to ferroptotic processes, both as a product of lipid peroxidation and as a propagator of further oxidative damage. It is one of the most reactive aldehydes generated during oxidative stress and readily forms adducts with cysteine residues on proteins, including enzymes that guard against lipid peroxides. Elevated ACR and related aldehydes (such as 4-hydroxynonenal and malondialdehyde) are commonly detected in cells undergoing ferroptosis, and they contribute to membrane destabilization and cell death. In our experiments, CYP exposure in vivo and ACR exposure in vitro each led to reductions in xCT and GPX4, signaling a loss of ferroptosis defense and a cellular environment shifted toward lipid peroxidation. Alb treatment preserved xCT and GPX4 levels in both settings, indicating that it prevented the upstream causes of ferroptosis, namely ACR and ROS accumulation. This preservation helps maintain the glutathione–GPX4 axis, thereby blocking ferroptotic cell death in the bladder. It should be mentioned that the reduced level of xCT/GPX4 in bladder lysate could also be derived from the loss of urothelial cells caused by acrolein. Because acrolein also downregulated xCT and GPX4 in culture, the observed decreases could be a combined results of cell loss and direct protein downregulation caused by acrolein. However, in either case, albumin treatment largely prevented the loss of these proteins, supporting a cytoprotective action.

An intriguing aspect of our study is the demonstration of the direct interactions between Alb and the toxic mediators of CYP therapy. Alb preferentially absorbs oxidative and electrophilic insults, acting as a sacrificial target. When Alb was present during ACR or H_2_O_2_ exposure, we observed an intriguing shift in the distribution of oxidative damage: cellular proteins were protected against carbonylation, whereas Alb itself became highly carbonylated. SDS-PAGE analysis of culture supernatants showed a prominent carbonylated band at ~66 kDa, corresponding to Alb, while cell lysates from the same samples were relatively spared. Complementary immunoblotting confirmed that ACR covalently adducts to Alb, leading to carbonyl formation; if the same reaction occurs in vivo, it is conceivable that Alb may act as a dominant reservoir for ACR, given its abundance in circulation. Indeed, Alb-ACR adducts have been previously reported and are considered a major form of ACR in body fluids [40]. Most circulating ACR rapidly becomes Alb-bound. We also showed that Alb undergoes specific thiol oxidation in the presence of H_2_O_2_, forming sulfenic acids at Cys-34 while concomitantly reducing H_2_O_2_ levels in solution. This thiol chemistry is well known as an aspect of Alb’s antioxidant function, and here we clearly demonstrated that it protects cells from H_2_O_2_-induced damage via this mechanism. Together, these findings support the generally accepted view that Alb protects cells from the effects of various reactive species. By acting as a “molecular sponge” for ACR and a scavenger for ROS, Alb spares essential cellular macromolecules, such as DNA, membrane lipids, and critical enzymes, from modification, thereby maintaining cellular viability and function.

While our results highlight ACR scavenging and ROS neutralization as key factors underlying Alb’s protective actions, Alb’s pleiotropic properties may also contribute to protection. Alb is known to bind transition metals (like iron and copper), which could help curtail Fenton chemistry that produces hydroxyl radicals from H_2_O_2_. Alb also transports a variety of hydrophobic molecules, including peroxidation-derived lipids and inflammatory mediators [41]. By sequestering such molecules, Alb could reduce their availability to participate in or amplify injurious processes. Moreover, Alb’s role in maintaining plasma oncotic pressure could also affect tissue edema and perfusion. This physical effect likely contributed to the markedly smaller bladder swelling seen in Alb-treated mice. Lastly, by neutralizing reactive species, Alb might indirectly suppress redox-sensitive inflammatory pathways. Oxidative stress can activate transcription factors (such as NF-κB and AP-1) and inflammasomes, which drive cytokine production and leukocyte recruitment. Reducing the oxidative stimulus with Alb could thus result in lower levels of pro-inflammatory cytokines and attenuated immune cell activation, complementing the direct chemical detoxification with an immunomodulatory benefit. While our study was not designed to dissect these auxiliary roles in detail, they likely act in concert with the primary ACR/ROS scavenging functions to produce the potent protection we observed.

Our finding that oral Alb administration can attenuate CYP-induced cystitis is intriguing and could have practical applications. Orally administered Alb is typically degraded in the gastrointestinal tract. How did feeding Alb replicate the effects of the systemic Alb delivery? This question remains to be addressed. One possibility is that a fraction of Alb or bioactive peptide fragments survives digestion and is absorbed, thereby augmenting the plasma pool of Alb or its functional thiols. Another possibility is that oral Alb provides an abundance of cysteine and other amino acids that bolster systemic antioxidant defenses, for example, by boosting glutathione synthesis in the liver and other tissues. Thus, the oral Alb likely acts by enhancing systemic defense rather than delivering intact protein to the body.

Additionally, oral Alb or protein supplements could modulate gut-derived factors that influence systemic inflammation or metabolism, indirectly protecting against CYP toxicity. The concurrent protective effect of dietary Alb supports the notion that nutritional interventions can enhance the body’s defense and detoxification capacity against ACR and, probably, other toxicants as well. Although the precise mechanisms remain to be determined, the oral approach has a clear translational advantage due to its simplicity and non-invasive nature. Patients could potentially take oral protein supplements as a prophylactic measure during chemotherapy to reduce the possible toxic side effects.

Although Alb is a plausible extracellular scavenger for ACR and diffusible oxidants, its in vivo site of action requires consideration. Under physiological conditions, intact Alb is largely excluded by the glomerular filtration barrier, making it unlikely that circulating Alb directly enters the urine in sufficient amounts to neutralize ACR within the bladder lumen. Nevertheless, several alternative routes could enable Alb to intercept ACR and H_2_O_2_ in vivo. First, Alb can react with reactive species in the systemic circulation and interstitial space, thereby lowering the overall burden of circulating electrophiles/oxidants and potentially reducing their delivery to the urinary tract. Second, CYP-induced urothelial injury is accompanied by inflammation and vascular leakage; extravasated Alb in the edematous bladder wall could therefore serve as a local “buffering” pool that preferentially reacts with ACR and H_2_O_2_ before they diffuse into cells, consistent with our observation that Alb shifts oxidative modifications away from cellular proteins. Third, tissue injury can compromise barrier integrity and increase protein flux from plasma into the bladder wall and, in severe cases, into the lumen; thus, Alb derived from the injured site via plasma exudation/hemorrhage could gain access to compartments where ACR is concentrated and directly neutralize it. Together, these considerations support a model in which Alb limits CYP cystitis primarily through extracellular detoxification in the circulation and, importantly, within the leaky, edematous bladder microenvironment rather than by simple neutralization of ACR in normal urine. This framework also provides a mechanistic rationale for why hypoalbuminemia may increase susceptibility to toxicity and why Alb supplementation could be protective, especially under conditions of inflammation-associated vascular permeability.

Our findings have several implications. First, they suggest that Alb levels may strongly influence a patient’s risk of chemotherapy toxicity. Many cancer patients have hypoalbuminemia due to malnutrition or inflammation, and low Alb is linked to worse side effects. Our results support the idea that when Alb is low, the body has less capacity to neutralize toxic ACR and oxidants, thereby increasing vulnerability. Alb supplementation could therefore help reduce risk in susceptible patients. Second, Alb may protect tissues in a manner different from that of typical antioxidants or anti-inflammatory drugs. Rather than mainly acting on late-stage free radicals or broadly suppressing inflammation, Alb appears to “trap” ACR and reactive oxidants, such as H_2_O_2_, early before they trigger widespread damage. Third, our study highlights an underappreciated in vivo function of Alb: detoxification and buffering in extracellular fluids. Alb that leaks into tissues during edema is often viewed only as a marker of vascular leakage, but our data suggest it may actively neutralize reactive toxins and oxidants in the local environment, helping protect cells from injury. Finally, Acrolein exposure can arise from various sources (diet, smoke, pollution, lipid peroxidation, etc.) and has been implicated in many diseases. Therefore, the role of endogenous albumin in binding and neutralizing acrolein may be important beyond the context of CYP. Because Alb is already FDA-approved, these findings may be readily translatable. The fact that oral Alb also showed benefit suggests a potentially convenient supportive approach to reduce CYP-related bladder injury.

The study also has limitations. Although we have clearly demonstrated a protective effect of Alb against CYP toxicity, the pharmacokinetics and dynamics of Alb–ACR interactions in vivo remain to be determined. It is also unclear whether albumin binds phosphoramide mustard (the active CYP metabolite), which could potentially reduce the drug’s anticancer effect. How to protect against CYP toxicity without compromising its antitumor efficacy is a question to be tackled in the future. Additionally, Alb was used as a pretreatment in this investigation; it is unclear whether it remains effective after model induction. Moreover, the detailed molecular mechanisms by which Alb influences intracellular events, such as ferroptosis, need to be clarified. Furthermore, it is also worth testing whether the protective effects of Alb could be extended to other toxic metabolites or drugs. Addressing these issues will promote the translation of current findings towards clinical applications.

## 5. Conclusions

In summary, our study characterizes Alb as a multifaceted protector against CYP-induced bladder injury. By sequestering the urotoxic metabolite ACR and scavenging ROS, Alb prevents oxidative damage and ferroptotic cell death in the bladder and attenuates the pathological activities of CYP cystitis. Given Alb’s safety profile and availability, these findings provide a strong rationale for its clinical use to mitigate chemotherapy-associated toxicities. More broadly, this work exemplifies how enhancing endogenous defense mechanisms, such as the detoxifying and redox-buffering functions of plasma proteins, can effectively protect tissues from injury caused by reactive drug metabolites and oxidative stress.

## Data Availability

The data is contained within the article.

## Supplementary Materials

The following supporting information can be downloaded at: https://www.mdpi.com/article/10.3390/biom16040536/s1, Figure S1: Western blot analysis of albumin in mouse serum and BSA using the anti-albumin antibody used in this study. Mouse serum samples (at the indicated dilutions) and bovine serum albumin (BSA, at the indicated concentrations; 20 μL each) were mixed with an equal volume of 2 × SDS sample buffer containing 50 mM DTT. After denaturation, 20 μL of each sample was loaded per lane on an SDS-PAGE gel. Proteins were separated by electrophoresis, transferred to PVDF membranes, and probed with a rabbit anti-albumin antibody (1:10,000; Protein tech: cat no. 16475-1-AP; Chicago, IL, USA) followed by an HRP-conjugated goat anti-rabbit secondary antibody. The membrane was then stained with EZ-Blue to visualize total protein; the albumin band (arrow, Alb) is visible at the expected molecular weight in both mouse serum and BSA, indicating an existence of cross-reaction.

## Abbreviations

The following abbreviations are used in this manuscript:

CYP	Cyclophosphamide
Alb	Albumin
ACR	Acrolein
H_2_O_2_	Hydrogen Peroxide
GPX4	Glutathione Peroxidase 4
xCT	Cystine/Glutamate Antiporter
ROS	Reactive Oxygen Species
BSA	Bovine serum Albumin
DMEM	Dulbecco’s Modified Eagle Medium
FBS	Fetal Bovine Serum
AM	Acetoxymethyl
PI	Propidium Iodide
MW	Molecular Weight
LDH	Lactate Dehydrogenase
-SH	Sulfhydryl
-SOH	Sulfenic Acid
HE	Hematoxylin Eosin
