# Supplementary material for: Albumin Protects Against Cyclophosphamide-Induced Hemorrhagic Cystitis by Scavenging Acrolein and Reactive Oxygen Species
Source: Biomolecules. 2026 Apr 3;16(4):536. doi: 10.3390/biom16040536 (PMC13113450; doi:10.3390/biom16040536)
Supplement: Supplementary file 1 [file biomolecules-16-00536-s001.zip › biomolecules-4185112-supplementary.pdf]

Supplementary Figure S1

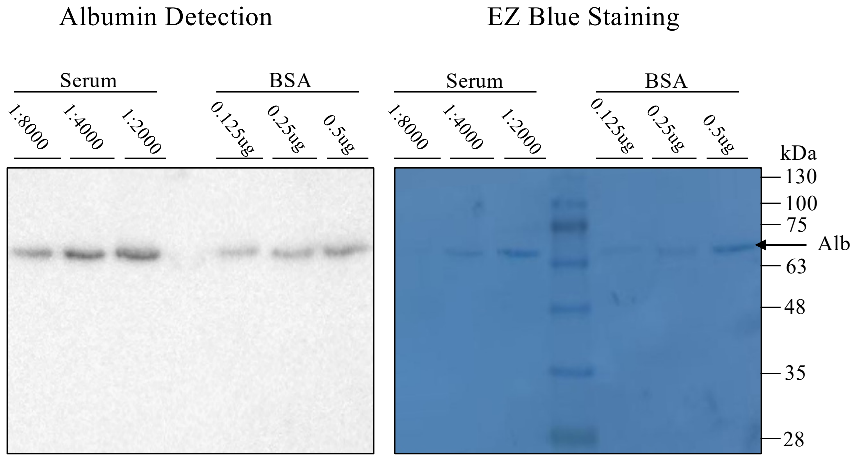

Supplementary Figure S1. *Western blot analysis of albumin in mouse serum and BSA using the anti-albumin antibody used in this study. Mouse s*erum samples (at the indicated dilutions) and bovine serum albumin (BSA, at the indicated concentrations; 20 µL each) were mixed with an equal volume of 2 × SDS sample buffer containing 50 mM DTT. After denaturation, 20 µL of each sample was loaded per lane on an SDS-PAGE gel. Proteins were separated by electrophoresis, transferred to PVDF membranes, and probed with a rabbit anti-albumin antibody (1:10,000 ; Proteintech: cat no. 16475-1-AP; Chicago, IL, USA) followed by an HRP-conjugated goat anti-rabbit secondary antibody. The membrane was then stained with EZ-Blue to visualize total protein; the albumin band (arrow, Alb) is visible at the expected molecular weight in both mouse serum and BSA, indicating an existence of cross-reaction.
